# Review of Droplet Printing Technologies for Flexible Electronic Devices: Materials, Control, and Applications

**DOI:** 10.3390/mi15030333

**Published:** 2024-02-28

**Authors:** Jiaxin Jiang, Xi Chen, Zexing Mei, Huatan Chen, Junyu Chen, Xiang Wang, Shufan Li, Runyang Zhang, Gaofeng Zheng, Wenwang Li

**Affiliations:** 1 School of Mechanical and Automotive Engineering, Xiamen University of Technology, Xiamen 361024, China; jjx@xmut.edu.cn (J.J.); chenxi0519up@163.com (X.C.); 35120190154074@stu.xmu.edu.cn (H.C.); wx@xmut.edu.cn (X.W.); zhang_runy@xmut.edu.cn (R.Z.); 2 School of Materials Science and Engineering, Xiamen University of Technology, Xiamen 361024, China; 19836950065@163.com; 3 School of Opto-Electronic and Communication Engineering, Xiamen University of Technology, Xiamen 361024, China; 35120201151504@stu.xmu.edu.cn (J.C.); sfli@xmut.edu.cn (S.L.); 4 Pen-Tung Sah Institute of Micro-Nano Science and Technology, Xiamen University, Xiamen 361102, China

**Keywords:** droplet printing, flexible electronic device, control, inkjet printing, electrohydrodynamic printing

## Abstract

Flexible devices have extensive applications in areas including wearable sensors, healthcare, smart packaging, energy, automotive and aerospace sectors, and other related fields. Droplet printing technology can be utilized to print flexible electronic components with micro/nanostructures on various scales, exhibiting good compatibility and wide material applicability for device production. This paper provides a comprehensive review of the current research status of droplet printing technologies and their applications across various domains, aiming to offer a valuable reference for researchers in related areas.

## 1. Introduction

Droplet arrays have garnered widespread attention in recent years within the fields of materials science, biology, and chemistry by precisely controlling the formation, arrangement, and manipulation of droplets [1,2,3,4,5,6,7]. The development of droplet array preparation technology has been facilitated by the rapid progress in microfluidic technology and a profound understanding of liquid behavior at the microscale [8,9]. Over the past decade, droplet array preparation has demonstrated substantial potential in fields such as optoelectronic displays [10,11], sensing and detection [12], and materials synthesis. For instance, in the field of biology, droplet arrays have been applied to study interactions between cells and biomolecules [13]. In materials science, droplet array preparation allows for precise control over small volumes of materials [14], including liquid metals [15], polymers [16], and organic semiconductors [17,18], enabling the fabrication of materials with specific properties. In the domain of chemistry, droplet array preparation contributes to the exploration of fundamental principles, such as chemical reaction kinetics [19].

The formation and control of droplets is the core of droplet preparation technologies. Currently, the predominant methods for droplet generation include dripping, jetting, and templating. Dripping involves regulating the generation of droplets by adjusting the velocity of the capillary tip and the surface tension between the droplet and substrate [20]. Jetting utilizes high-speed liquid streams to form droplets in the air, while templating utilizes prefabricated microhole templates to arrange droplets in an orderly manner. Each of these methods presents advantages and limitations in the preparation of droplet arrays, allowing researchers to select suitable methods based on specific requirements.

The scope of applications for droplet array preparation continues to broaden. Researchers are not only exploring efficient and controllable methods for droplet generation, but are also focusing on optimizing the performance of droplet arrays in practical applications. For instance, in the field of biosensing [21,22], optimization of the preparation process for droplet arrays can enhance the binding efficiency of droplets with biomolecules, thereby achieving sensitive and rapid detection. In materials science, droplet arrays can be utilized to fabricate ordered structures of inorganic materials [23,24] or organic optoelectronic materials [25,26,27,28] that can subsequently be used to investigate the relationship between their macroscopic properties and microscopic structures.

This review compares different printing technologies and discusses the control techniques and areas of application for precise droplet printing.

## 2. Typical Generation Mechanisms of Droplet Printing

### 2.1. Inkjet Printing

**Figure 1:**
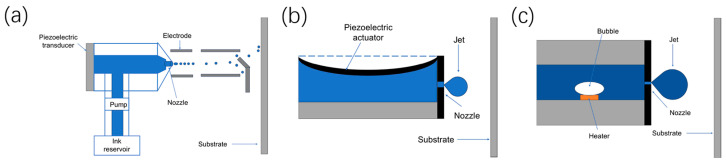
Schematic diagram of typical inkjet printing methods: (**a**) Schematic diagram of continuous inkjet printing. (**b**) Schematic diagram of piezoelectric inkjet printing. (**c**) Schematic diagram of thermal inkjet printing.

Inkjet printing technology is currently the most widely used non-contact electronic printing method. This technique is realized by the ejection of ink droplets onto a collection plate through a series of nozzles. Inkjet printing consists of two different ejection modes: continuous inkjet (CIJ) and drop-on-demand (DOD). A diagram illustrating the principle of continuous inkjet printing is shown in Figure 1a. In continuous inkjet printing, a continuous cylindrical ink jet is ejected from the nozzles, after which the stream is broken up into ink droplets by a stimulating jet, where the size and spacing of the ink droplets can be controlled. Printing information is formed by controlling the charges on the nozzles to generate ink droplets with and without charges. The spatial electric field alters the flight path of the ink droplets, directing the ink droplets onto the collection plate to create character/graphic records. Ink droplets not used for printing are recollected through a conduit. 

In contrast to CIJ, in DOD printing, ink droplets are expelled only when needed, with the ejection of droplets from each nozzle governed by a triggering signal controlled by an actuator within the nozzle. In this way, it can be divided into different modes according to the driving source. Piezoelectric inkjet printing and thermal inkjet printing are the most common inkjet printing methods and are depicted in Figure 1b,c. In piezoelectric inkjet printing technology, a transducer is installed on the nozzle to control the contraction and stretching of the piezoelectric element through changes in microvoltage, which offers advantages in terms of accuracy, speed, and versatility. However, it also presents certain drawbacks such as cost, limitations on the types of fluids used, and maintenance requirements. In thermal inkjet printing, a resistance heater is used to rapidly heat the ink in the capillary tube to its boiling point so it evaporates, resulting in tiny steam bubbles. After the steam bubbles expand and rupture, droplets are formed at the top of the capillary tube and sprayed out. However, its operational principle make the nozzle susceptible to impurities and deposits, resulting in clogging and affecting print quality. Additionally, the print lifespan of thermal inkjet heads is constrained by the material and heat resistance of the nozzles, leading to a relatively shorter print lifespan. 

### 2.2. Electrohydrodynamic Printing

**Figure 2:**
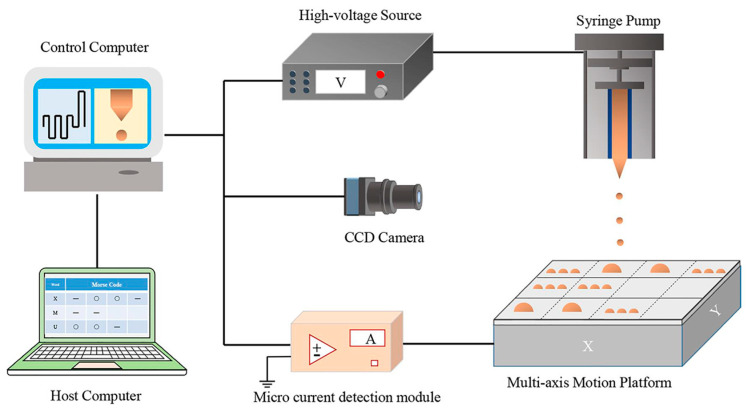
Schematic diagram of electrohydrodynamic printing. Reproduced with permission from [40] under the Creative Commons CC BY license.

Different from traditional piezoelectric and thermal inkjet printing, as well as other printing methods, electrohydrodynamic printing involves applying a specific voltage on the nozzle such that a high-voltage electric field is generated between the nozzle and the substrate. Through the action of electric field forces, the surface tension of the droplet at the apex of the nozzle is overcome. Then, the droplets are ejected from the nozzle to deposit at predefined positions, forming the desired pattern with high resolution on the targeted substrate, as shown in Figure 2. Electrohydrodynamic printing can be applied in the manufacture of thin-film transistors [29,30,31], protein microarrays [32], DNA microarrays [33], block copolymer thin films with self-assembly effects [16], optical devices, conductive electrodes [34,35,36,37] etc., and can be used in technologies such as cell culture, quantum dot displays [38,39], and 3D structural printing.

Several research groups have been engaged in the investigation of droplet printing technologies, and a large number of functional materials with various properties were printed into different structures to meet specific application requirements. Typical methods for droplet printing are listed in Table 1.

## 3. Liquid Modification for Printing Materials

In droplet printing technologies, the performance of devices is determined by the functional inks used. The use of organic and inorganic printing materials significantly expands the applications of droplet preparation. Characteristics such as ink viscosity, conductivity/surface charge density, and surface tension greatly influence inkjet formation, interfacial interactions between the ink and the collection plate, and droplet drying. Therefore, using different ink materials greatly assists in achieving high-precision droplet positioning and shaping.

**Figure 3:**
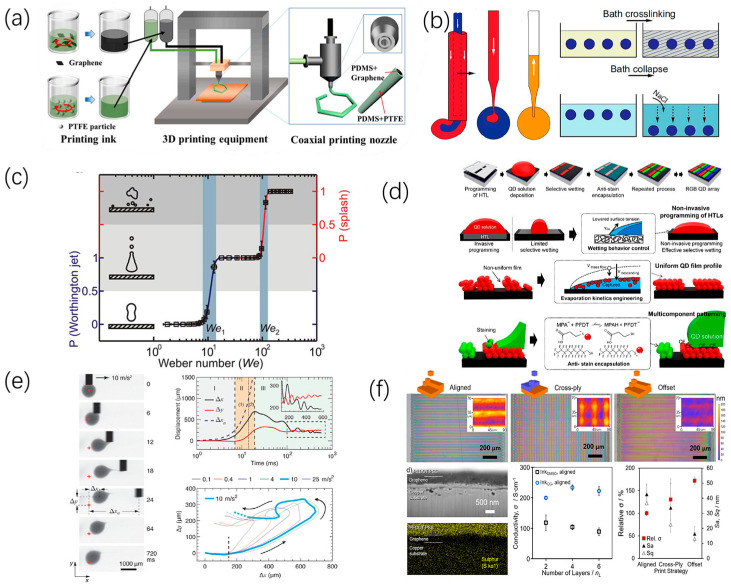
Liquid modification of printing materials: (**a**) Schematic diagram of configuration of the solution for coaxial stretchable smart 3D printing process fibers. Reproduced with permission from [55], published by Elsevier, 2021. (**b**) Schematic diagram of embedded droplet printing. Reproduced with permission from [56] under the Creative Commons CC BY license. (**c**) Description of droplet dynamics, depicting the impact speed and pillar influence schematic. Reproduced with permission from [57], published by the American Chemical Society, 2014. (**d**) Diagram of the principles of non-invasive programmed-patterning (NIPP). Reproduced with permission from [58], published by the American Chemical Society, 2022. (**e**) Trajectory of ink droplets in the separation phase. Reproduced with permission from [47], published by Elsevier, 2023. (**f**) Schematic of printing strategy for produced thin films. Reproduced with permission from [52] under the Creative Commons CC BY license.

Guo et al. [54] used non-polar solvents to regulate inkjet printing technology, achieving inkjet-printed nano-particle self-assembled continuous lines with adjustable morphology, high resolution of printed microarrays, and continuous line widths of <5 μm and 10 μm. Chen [55] mixed graphene and PDMS to obtain printable inks, with graphene also used to manipulate rheological behavior to meet the requirements of extrusion-based printing, as shown in Figure 3a. Nelson [56] designed an embedded droplet printing system using the special properties of yielding stress fluids, achieving highly precise control at microscale for customization of fluid droplet generation and handling, as shown in Figure 3b. Aria [57] reported that adding active oligomeric surfactants to the solution results in more uniform diffusion of impacting droplets. Splashing during printing is suppressed, and diffusion is very uniform; this approach thus holds great potential for high-resolution printing requirements, as shown in Figure 3c. Song [58] introduced a method to pattern quantum dot arrays by controlling the evaporation and diffusion of microdroplets on the substrate; this method was used for preparing high-resolution full-color quantum dot arrays, thus achieving non-invasive direct patterning of quantum dots, as shown in Figure 3d. Zhu [47] used a mixture of mineral oil and red dye as ink and achieved efficient printing by adjusting flow rate and ink concentration, as shown in Figure 3e. Rivers [52] developed a method for preparing stable large-area droplet-demand conductive polymer inks for 3D printing of electronic products, using a bio-renewable co-solvent to address the poor stability and large-area droplet-demand issues associated with conductive polymer inks, as shown in Figure 3f.

## 4. Control Methods for Droplet Printing

### 4.1. Generation Control for Droplet Printing

In order to achieve precise control over the deposition of droplets, it is necessary to experimentally compare the effects of various factors on droplet deposition. Understanding the influence of multiple factors on the deposition process is essential. Therefore, this section primarily focuses on summarizing and reviewing the research progress related to the deposition mechanisms of ink droplets, patterned deposition, deposition positioning control, and the engineering applications of droplet printing technologies.

#### 4.1.1. Assisted-Field Control

A multi-physics field was applied in the droplet printing process, often leading to inaccurate droplet deposition and the formation of satellite droplets during the production, flight, and oscillation of droplets in the space between the nozzle and the collection plate. Therefore, achieving precise positioning and control of charged droplets is crucial. Various assisted fields have been introduced into droplet printing technologies, including assisted electrical fields, assisted gas fields, assisted magnetic fields and assisted laser fields. Through appropriate modification of the spatial field distribution, the formation and deposition accuracy of droplets can be promoted.

**Figure 4:**
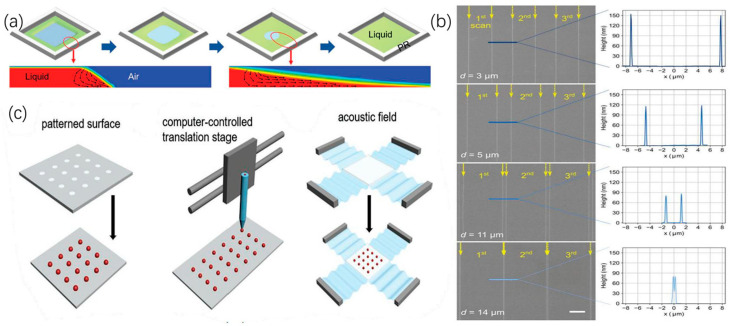
Control method for droplet printing with assisted field: (**a**) Schematic diagram of C8-BTBT thin-films preparation process with a hydrophilic external field scaffold. Reproduced with permission from [59], published by John Wiley and Sons, 2021. (**b**) Schematic diagram of microdroplets during printing based on sonic field control. Reproduced with permission from [53], published by John Wiley and Sons, 2020. (**c**) Schematic diagram of droplet printing by magnetic and acoustic field control. Reproduced with permission from [60], published by John Wiley and Sons, 2023.

Peng [42] proposed a method to rapidly fabricate 3D microelectrode arrays by combining inkjet printing and laser ablation technology, enabling simple, fast, and cost-effective production of 3D microring electrode arrays. Fang [59] designed a method for inkjet printing that relies on the assistance of hydrophilic microscaffolds, enabling precise patterning of C_8_-BTBT thin films with large single-crystal domains by strictly controlling the deposition position of LC materials during inkjet printing, as shown in Figure 4a. Chen [53] designed a method that uses the abnormal electric-field distribution generated by inter-nozzle crosstalk of adjacent printing nozzles to control the transport and arrangement of ink droplets. The unusual electric-field distribution generated by crosstalk between adjacent dispenser holes can be used to intricately control the microjet path of the ink, thereby enabling on-demand control of shape, position, and material composition in the 3D printing of nanostructures. Compared to traditional serial methods, this parallel method significantly improves productivity while achieving nanoscale printing of multiple materials, as shown in Figure 4b. Chai [60] introduced a method for capturing and manipulating small objects using sound waves generated by piezoelectric materials. Piezoelectric actuators produce sound waves at different frequencies under varying voltages, such that control over the frequency and amplitude of the sound waves can be used to successfully capture and manipulate small droplets of varying sizes and shapes. Chai [60,61] utilized the interaction between a magnetic field generated by an electromagnetic coil and sound waves to capture small objects, designing an electromagnetic-driven acoustic capture device that, by altering the coil’s current and frequency, achieves capture and manipulation of small objects. This method boasts high capture efficiency and precision, as shown in Figure 4c.

#### 4.1.2. Electrical Excitation

In traditional inkjet printing methods, the droplet is triggered by an excitation signal at a specific frequency. However, due to the hysteresis of liquid rheology that arises from the action of surface tension and viscous forces, the accurate control of droplet generation remains a problem to be solved. Several works have reported acceleration of the fluid response by application of an external electrical excitation.

**Figure 5:**
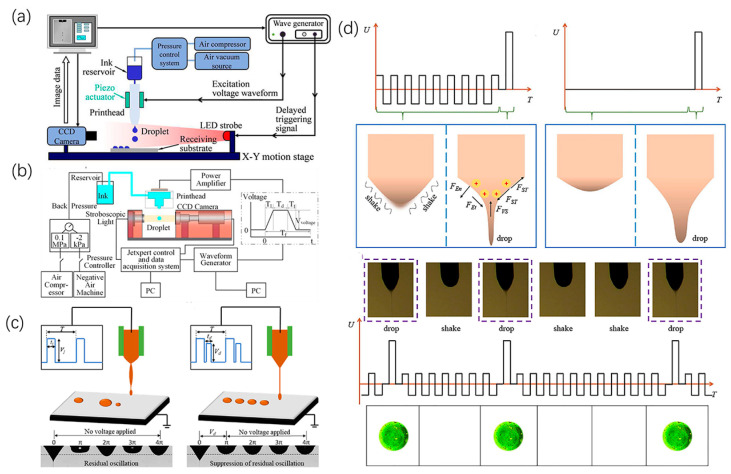
Control method for droplet printing with electrical excitation: (**a**) Schematic diagram of piezoelectric on-demand inkjet printing device. Reproduced with permission from [48], published by Elsevier, 2021. (**b**) Schematic diagram of the device for preparing droplets by driving voltage. Reproduced with permission from [49], published by Elsevier, 2022. (**c**) Schematic of electrohydrodynamic printing with optimized waveforms. Reproduced with permission from [62], published by Elsevier, 2022. (**d**) Schematic diagram of a fast on-off controlling electrohydrodynamic printing system. Reproduced with permission from [40], published by Elsevier, 2022.

Lohse [61] introduced a method for on-demand inkjet printing using piezoelectric droplets whereby the deformation of piezoelectric ceramic materials results in a change in ink volume within the pressure chamber, thereby generating pressure waves propagating towards the nozzle and allowing droplet formation at the nozzle. When the droplet forms, the pressure must be sufficient to expel the droplet toward the recording medium. Zhao [48] extensively discussed the influence of excitation waveforms such as pulse-width modulation, frequency modulation, amplitude modulation, and sinusoidal excitation on the performance of conductive polymer inks in inkjet printing. Optimizing different excitation waveforms can improve printing quality, speed, and stability, providing valuable references for practical applications, as shown in Figure 5a. Yang [49] optimized the drive waveform using a multi-pulse interleaving modulation method, leveraging the orthogonal interleaving effect inside the nozzle as a control variable. This adjustment of the driving voltage waveform achieved high-precision droplet printing, as shown in Figure 5b. Li [62] described a method for suppressing residual oscillations through waveform optimization to achieve stable on-demand droplet printing. Through the adjustment of voltage, frequency, and other printing parameters, the amplitude of meniscus vibration was reduced, significantly enhancing the stability and uniformity of droplet printing, as shown in Figure 5c. Chen [40] introduced a novel electrohydrodynamic microdroplet rapid-switching-control technology. By the application of alternating induced voltage, the suspended droplet interface is swiftly breached, resulting in a significant reduction of the impulsive current from 527.2 to 50.14 nA and thus markedly mitigating its adverse impact on jet stability. Additionally, controllable and large-scale formation of microdroplets is achieved, with each droplet’s structure being independently regulated, as shown in Figure 5d.

#### 4.1.3. Printing Quality Modeling 

With the rapid development of artificial intelligence, there is a trend of innovation in droplet printing. Printing information including the jet image, the electric current, the liquid rheology, the structure morphology are detected and entered into the intelligence control system to predict the droplet printing behavior. In this way, the requirement for operational skill is largely reduced, contributing to the accelerated development of industrial applications for droplet printing technologies. 

**Figure 6:**
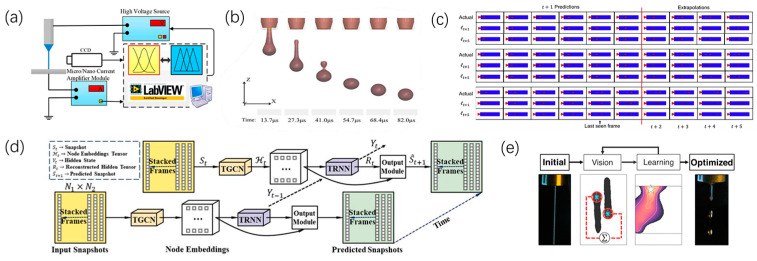
Control method for droplet printing with printing quality modeling: (**a**) The closed-loop feedback system based on current detection and image recognition with electrohydrodynamic direct-writing. Reproduced with permission from [63] under the Creative Commons CC BY license. (**b**) Snapshots of temperature contour of inkjet process simulation result. Reproduced with permission from [41], published by Elsevier, 2023. (**c**) Preceding predictive results of frame sequences from the video of the droplet formation process. Reproduced with permission from [50], published by Elsevier, 2020. (**d**) Schematic diagram of the NET scheme applied to tensor time series. Reproduced with permission from [67], published by Elsevier, 2023. (**e**) Bayesian optimization and computer-vision feedback-loop diagram. Reproduced with permission from [17], published by the American Chemical Society, 2017.

Zheng [63,64,65,66] developed a closed-loop feedback system based on current detection and image recognition to enhance jet stability and microstructure-deposition accuracy. This research provides a promising approach for designing optimized control algorithms and implementing closed-loop control systems, thereby contributing to improved jet stability and the expedited application of electrohydrodynamic direct-writing (EDW) technology, as shown in Figure 6a. Soon Wook Kwon [41] improved the predictive accuracy of the material printing process by introducing physical constraints into neural networks, as shown in Figure 6b. Huang et al. [50] studied the evolution behavior and process dynamics of ink droplets in the inkjet printing process using unsupervised learning methods. By using video data instead of images to study droplet evolution during inkjet printing, the experimental results demonstrated the high accuracy of the proposed method in predicting droplet evolution and understanding the dynamics of the inkjet printing process, as shown in Figure 6c. Segura [67] studied the evolution of droplet behavior with different materials and process parameters through tensor time-series analysis of experimental data. The author successfully predicted the evolution behavior of droplets with different materials and process conditions using this method, as shown in Figure 6d. Siemenn [17] proposed a method for optimizing the droplet-generation process using Bayesian optimization algorithms, effectively improving the efficiency and accuracy of the droplet-generation process and thereby enhancing the performance of droplet arrays, as shown in Figure 6e. Mea [68] utilized a glass capillary microfluidic device to achieve programmed entrapment of droplets. Through this method, the properties of elastomers could be dynamically adjusted in real time during the printing process, allowing extruded ink to be regulated by using droplet entrapments during printing and enabling on-demand tuning of 3D printed elastomers. Bucciarelli [69] reported a study using a statistical method, namely design of experiments (DOE), to optimize the inkjet printing parameters for a nanoparticle-based silver (Ag) ink. This method showed the interplay between the waveform parameters, and the definition of optimal drop reproducibility, the achievement of the optimal resolution. These equations can be used as a tool to directly tune the properties of the printed dot by modifying the waveform parameters.

### 4.2. Deposition Control for Droplet Printing

During the droplet printing process, control of the morphology of the deposited droplets is of great significance, as it directly affects the resolution and performance of the desired patterns. When the generated droplets reach the substrate, the interaction effect easily leads to phenomena such as the coffee-ring effect, spread, or splashing, which are affected by the properties and morphology of the target substrates. Therefore, the performances of the substrates have been investigated in relation to control of the droplet deposition process, with the aim of obtaining high-resolution droplets.

#### 4.2.1. Substrate Modification

The properties of substrates, including the substrate chemical composition, the surface temperature, and the surface roughness, are all important factors that determine the curing behavior of the ink and the flexibility of the equipment. Several strategies have been reported that can adjust the shape of solution droplets by modifying the properties of the substrate. 

**Figure 7:**
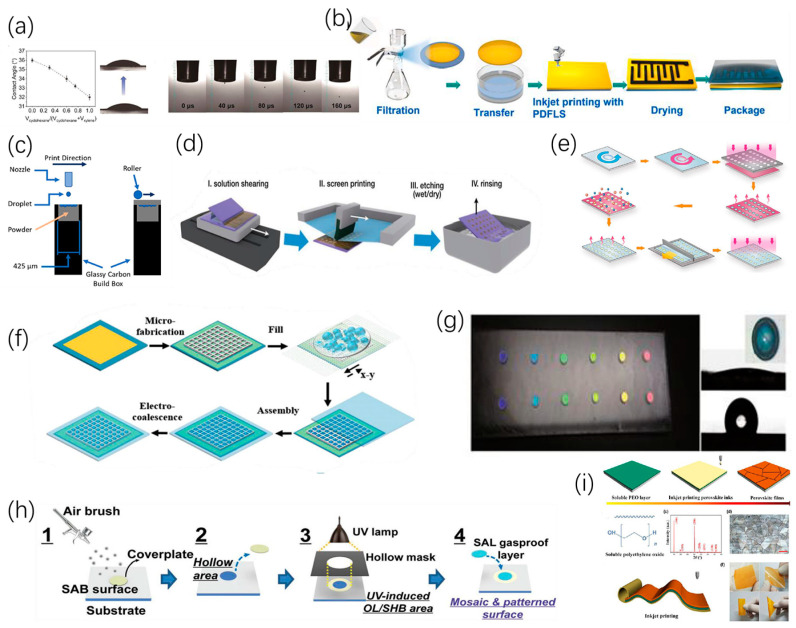
Control method for droplet deposition: (**a**) Formation of droplets on the surface of pure cyclohexane, xylene, and PDMS film before contact. Reproduced with permission from [54], published by John Wiley and Sons, 2023. (**b**) Schematic diagram of electrode manufacturing in inkjet printing. Reproduced with permission from [72], published by Elsevier, 2022. (**c**) Schematic diagram of pre-wetting powder bed printing. Reproduced with permission from [73], published by Elsevier, 2023. (**d**) Schematic diagram of the printing and patterning process using large-area organic high-crystal arrays. Reproduced with permission from [74], published by John Wiley and Sons, 2020. (**e**) MLA manufacturing process based on selective wetting. Reproduced with permission from [75] under the Creative Commons CC BY license. (**f**) ECDA chip-manufacturing process schematic: photolithography of micropores on ITO glass coated with Hyflon, emulsion filling, chip assembly and sealing, and micropore-constrained droplet electropolymerization. Reproduced with permission from [76], published by John Wiley and Sons, 2023. (**g**) Photography image of the PC sensor with different colors of PC dots. Reproduced with permission from [78], published by John Wiley and Sons, 2015. (**h**) Manufacturing method for mosaic and patterned surfaces. Reproduced with permission from [79], published by John Wiley and Sons, 2023. (**i**) Schematic diagram of controlled printing of large compact perovskite thin films. Reproduced with permission from [43], published by Springer Nature, 2021.

Guo [70] used zinc acetate dihydrate as a raw material for preparing particle-free ZnO functional ink. After inkjet printing on the PI flexible substrate and curing at 300 °C for 30 min, the pattern surface is smooth and clear and the outline is clear. Dan [71] claimed that the coffee-ring effect could be effectively reversed via cooling down the temperature of substrate. Guodan [54] promoted droplet retraction and controlled droplet coalescence and drying by using a polydimethylsiloxane (PDMS)-coated glass substrate, achieving high-resolution inkjet printing of microarrays, as shown in Figure 7a. Sun [72] reported that the simple pre-deposition of an ethanol layer enabled a series of procedures, including homogenization, solvent exchange, post-stretching, and air drying, thereby uniformly depositing densely structured graphene nanosheets and effectively limiting and eliminating the coffee-ring effect during inkjet printing, as shown in Figure 7b. Inkley [73] successfully used triethylene glycol for pre-wetting the powder bed before printing, significantly expanding the range of droplet spacing to produce continuous lines, as shown in Figure 7c. Duan [74] significantly altered the fluid drying kinetics by adding surfactants during solution printing and increasing the contact-line friction between the aqueous solution and the underlying non-wetting organic crystalline film. As a result, centimeter-level highly-arranged arrays of organic crystals were successfully prepared on different substrates, as shown in Figure 7d. Liu [75] reacted large-scale droplet arrays with controlled curvature by selectively modifying the surface using tunable oxygen plasma, promoting precise patterns by adjusting chemical contrast; they also used droplet dosage modification to achieve precise adjustment, as shown in Figure 7e. Feng [76] proposed and verified an efficient, high-throughput method for the rapid preparation of uniform droplet arrays induced by an electric field in multiple emulsion droplets within micropores. Polydisperse emulsions were prepared through mechanical stirring and then filling them into hydrophobic micropores through screen printing. As a result, driven by an alternating electric field, emulsion droplets restricted to the same micropore migrated and coalesced pair-wise into large droplets in individual micropores, forming regularly arranged droplets in the micropore array, as shown in Figure 7f. Kahng [77] reported a method to control single droplet behavior through strong capillary forces between the tip and the substrate, further improving printing accuracy.

#### 4.2.2. Substrate Pre-Patterning

In addition to the property modification of substrates, several research groups have been engaged in the design of pre-patterned substrates that can be used to guide the deposition morphology of printed droplets.

Hou et al. [78] also reported a printing strategy developed by directly manipulating droplet behavior on modified substrates, preparing hydrophilic and hydrophobic patterns, and then transferring intelligent material-based droplets onto the patterns, achieving patterned droplet printing, as shown in Figure 7g. Jiao [79] developed a method to generate mutually independent and almost non-volatile capsule droplet arrays using an innovative mosaic pattern surface, achieving in the array an evaporation inhibition 1712 times that of naked droplets and obtaining mutually independent droplet arrays, as shown in Figure 7h. Gu [43] proposed a method to achieve good control of the nucleation and crystal growth of perovskite thin films by introducing a soluble polyethylene oxide (PEO) layer during inkjet printing, allowing large-scale printing of perovskite thin films with high-resolution patterning, providing the possibility of developing flexible photodetectors, as shown in Figure 7i.

## 5. Application

Droplet printing technology has a wide range of applications in many fields, including biomedical, electronics, materials science [20], and nanotechnology. Especially in the field of micro flexible electronic advices [18], droplet printing technology has unique advantages in preparing micro-level structures [80], making it suitable for fields such as optoelectronic displays [81] and micro/nano system integration. The microsystem compatibility of this technology makes it an ideal choice for manufacturing various microdevices such as microsensors, microreactors, and flexible sensors [21,39].

### 5.1. Photoelectric Display

The use of droplet printing technology for the preparation of quantum dot films addresses the limitation of traditional printing methods in achieving fine patterns. The exceptional tunability of perovskite materials has garnered widespread attention in the field of optoelectronic displays. This approach enables the formation of required patterns without the need for templates and metal shadow masks.

**Figure 8:**
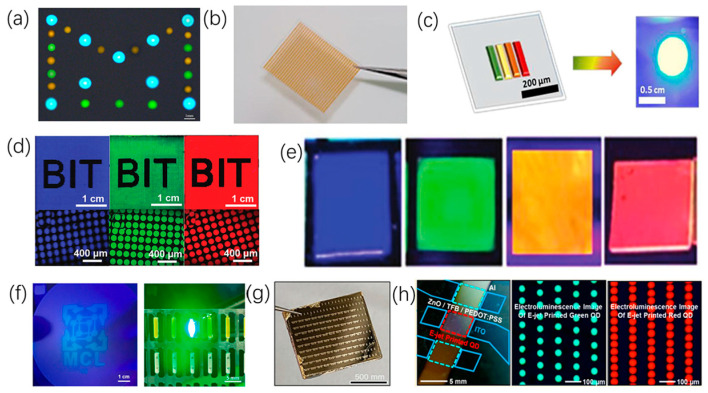
Photoelectric display: (**a**) Core-shell droplets of different colors and sizes. Reproduced with permission from [81] under the Creative Commons CC BY license. (**b**) Calcium titanate pattern printed on PET. Reproduced with permission from [43], published by Springer Nature, 2021. (**c**) Multi-color luminescent inorganic perovskite nanocrystals printed for the fabrication of white light-emitting devices. Reproduced with permission from [82], published by Elsevier, 2020. (**d**) Fluorescent image of perovskite quantum dot samples. Reproduced with permission from [83], published by John Wiley and Sons, 2019. (**e**) CsPbX3 thin film with different halide components under 365 nm UV light. Reproduced with permission from [84], published by John Wiley and Sons, 2019. (**f**) Device prepared by printing CsPbBr3/Cs4PbBr6 ink under UV light. Reproduced with permission from [85], published by Elsevier, 2021. (**g**) Photograph of a NOT gate circuit array based on patterned C8-BTBT LC film. Reproduced with permission from [59], published by John Wiley and Sons, 2021. (**h**) Uniform quantum dot array printed for quantum dot LED using E-jet. Reproduced with permission from [86], published by the American Chemical Society, 2015.

Chen [81] utilized the coaxial electrohydrodynamic printing technique, integrating real-time microcurrent signals with the behavior characteristics of core-shell droplets. This approach not only resulted in an understanding of the interfacial behavior of the droplets to be analyzed at the nozzle, but also clarified the process of microcurrent-induced droplet formation during core-shell processing. The study provides valuable insights for achieving high resolution in core-shell droplet printing, as shown in Figure 8a. Gu et al. [43] achieved excellent control of perovskite nucleation and crystal growth during the inkjet printing process by introducing a soluble polyethylene oxide (PEO) layer. The perovskite thin film can be readily printed on a large scale with high-resolution patterning. Perovskite thin film optical detectors exhibited a responsivity of up to 1036 mA/W and maintained over 96.8% of the initial photocurrent after 15,000 consecutive bending cycles, as shown in Figure 8b. Altintas [82] printed synthetic perovskite nanocrystals (PNC) with high photoluminescent quantum yields using electrohydrodynamic printing, resulting in controllable PNC patterns in different colors. This result led to the creation of a high-quality white LED with excellent luminance performance and stability, as shown in Figure 8c. Zhong [83] printed perovskite quantum dot film patterns on different polymer substrates using in-situ inkjet printing technology. By varying the substrate temperature, controlled adjustments of droplet size and contact angle were achieved, resulting in perovskite quantum dot film array dot lattice sizes of approximately 110 μm, as shown in Figure 8d. Tang [84] achieved high-resolution full-color perovskite quantum dot film patterns using electrohydrodynamic printing. Through process parameter optimization, stable printing of perovskite quantum dot film dot arrays with diameters less than 5 μm was attained, as shown in Figure 8e. Liu [85] printed ink on LED chips sized 9 × 45 mils, yielding a dried ink-layer thickness of 351 μm, resulting in color-converting mini-LED chips that maintained 55% of luminance intensity after operating for 116 h, as shown in Figure 8f. Fang [59] demonstrated a simple method that combines inkjet printing and melt-processing techniques to prepare patterned liquid crystal (LC) films, aiming to produce high-performance organic integrated circuits. The inverter based on patterned LC films exhibited a high gain of up to 23.75 and a noise margin exceeding 81.3%, paving the way for the production of high-performance organic integrated devices due to the excellent universality of the patterned process and the high quality of the obtained films, as shown in Figure 8g. Kim [86] achieved finely patterned quantum dots with sub-micron lateral resolution and adjustable thickness using electrohydrodynamic printing. The uniform quantum dot pattern array, composed of different quantum dot materials, enabled the fabrication of a series of outstanding optoelectronic devices. Additionally, quantum dot LEDs were obtained using electron-beam-deposited quantum dot patterns, with red and green quantum dot pixel resolution comparable to that of commercial displays, as shown in Figure 8h.

### 5.2. Micro/Nano Electronic System Components

Droplet printing is a flexible manufacturing technology with excellent material compatibility that can be used to prepare microstructures on different substrates with various physical characteristics and in different shapes. In this way, it has been a mainstream technology in the integration manufacturing of micro/nano system devices.

**Figure 9:**
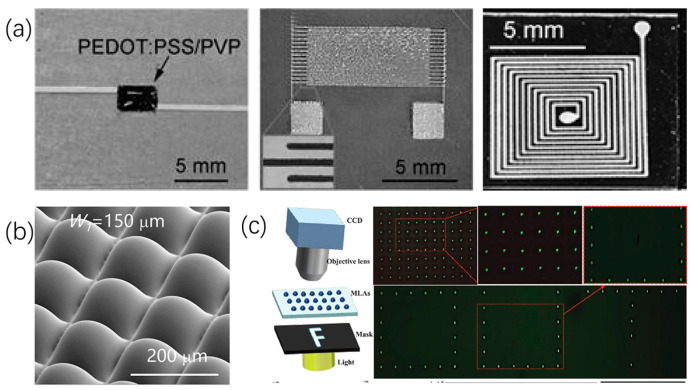
Micro-/nano-system components: (**a**) passive electrical components. Reproduced with permission from [87], published by Springer Nature, 2012. (**b**) Tailored Microlens Arrays. Reproduced with permission from [88], published by Elsevier, 2020. (**c**) The focal points of the MLAs. Reproduced with permission from [89], published by John Wiley and Sons, 2021.

Wang [87] successfully fabricated micron-scale-resolution conductive silver patterns using the electrohydrodynamic printing method and further demonstrated the production of several passive electrical components such as thin film resistors, fork-shaped capacitors (6 pF), and spiral inductors (0.6 μH), as shown in Figure 9a. Li [88] proposed a new method for preparing self-aligned microlens arrays using multi-functional electrohydrodynamic printing. This process, by regulating the mode of the confined region and the volume of droplets in each region, generated microlens arrays with different bottom shapes, aperture sizes, aspect ratios, and filling factors. The details of the impact on light extraction were discussed. Finally, a self-aligned MLA with a filling factor of up to 99.3% achieved 49% enhancement in light extraction, demonstrating its enormous potential for OLED light extraction, as shown in Figure 9b. Su [89] achieved an increase in the numerical aperture of microlens arrays from 0.18 to 0.53 through surface-modified polymer’s contact angle. By combining printing parameters with PDMS nano-film modification, various high numerical aperture microlens patterns were obtained through electrohydrodynamic printing. Projection experiments demonstrated that the microlens array exhibited uniformity and excellent optical performance. Additionally, the fabricated microlens array could generate virtual images with magnification as high as 1.72×, as shown in Figure 9c.

### 5.3. Integrated Sensors 

Given their small feature size and large specific surface area, microstructures prepared by droplet printing technologies have exhibited notable advantages in the production of sensors.

**Figure 10:**
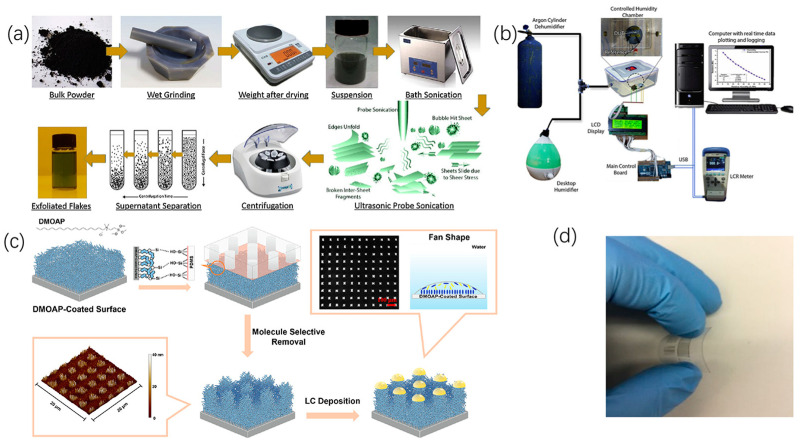
Integrated sensors: (**a**,**b**) Step-by-step liquid mechanical exfoliation process for preparing a 2D suspension of MoS_2_ flakes in ethanol; detailed schematic of an automatic humidity- and temperature-measuring device. Reproduced with permission from [92], published by Elsevier, 2020. (**c**) Schematic of the fabrication of LC microdroplet arrays. Reproduced with permission from [93], published by Elsevier, 2023. (**d**) Printed microelectrode array on PET film with flexibility. Reproduced with permission from [94], published by Elsevier, 2017.

Li [90] deposited ZnO thin films using an electrospray method and produced an alcohol gas sensor, exhibiting good repeatability and response stability in the target gas. Wang [21] introduced a method for preparing gel pressure sensors using hydrophobic/hydrophilic patterned surface. Through optimization of the array configuration of the sensor, an uneven conductive gel array was fabricated. The array exhibited high sensitivity (0.29 kPa^−1^ in the 0–30 kPa pressure range) and maintained a sensitivity of 0.13 kPa^−1^ in the 30–100 kPa range. Zhang [91] demonstrated a droplet laser array with integrated microfluidics on a silicon chip, generating and controlling four individual droplet optical cavities using a 2 × 2 nozzle array. Droplet arrays ranging in diameter from 115 to 475 μm could be generated, removed, and regenerated as needed, promising the development of miniature light sources and biological and chemical sensors. Yousaf [92] presented a temperature-compensated integrated sensor wherein the sensor electrodes were fabricated using electrohydrodynamic printing technology and the active layer of the humidity sensor was covered by an electrospray-deposited polymer. The humidity sensor’s active layer was made of a novel composite of polyethylene oxide (PEO) and 2D molybdenum disulfide (MoS_2_) flakes, achieving high sensitivity (85 k Omega/%RH) and almost linear responsiveness over a wide detection range (0–80% RH) of relative humidity, As shown in Figure 10a,b. Chang [93] introduced a molecular patterning technique with spatial control for spatial control molecular patterning for preparing liquid crystal (LC) microdroplet arrays on glass substrates. This technique utilized an oxygen plasma-activated PDMS stamp to remove pre-coated dimethyloctadecyl[3-(trimethoxysilyl)propyl] ammonium chloride (DMOAP) molecules from the glass substrate, resulting in surfaces with complementary patterns and specific hydrophobicity. When LC molecules were introduced onto these produced molecular patterns, LC microdroplet arrays with uniform droplet sizes and positional order were formed, as shown in Figure 10c. Qin [94] manufactured a capacitive touch sensor by using electrohydrodynamic printing technology to print AgNPs onto PET film. A high-resolution microelectrode array with a resolution of up to 15 μm was successfully developed. Additionally, the sensor exhibited high flexibility, high sensitivity, and a short response time (~30 ms), making it further suitable for use in microcapacitance, inductance, or electrode arrays as a flexible display, as shown in Figure 10d.

## 6. Summary and Future Prospects

Droplet printing technologies have shown excellent advantages in the integrated manufacturing of micro-/nano-scale flexible electronic devices. However, given the small feature size and fast printing speed, the stable generation and deposition of droplets are still key challenges for its applications. Several works have focused on control strategies to promote the printing accuracy of droplet arrays, including apparatus design, liquid interface control, electrical and physical field regulation, and an intelligent system, which has been applied in flexible electronics to promote integration and device performance.

Although much progress has been made in the droplet printing technologies and its application in flexible devices, there are still many challenges to be investigated in future works:

(1) Droplet printing technologies are still mostly limited to the laboratory research. Given the excellent performance of printed structures, there is urgent demand for the spread of novel printing methods to industrial fields. Parallel multi-channel printing is expected to be an effective way to realize the high-throughput production of droplets [95,96]. However, the accurate and controllable synchronous deposition of droplet arrays at high resolution is still a serious challenge. More intensive study focused on the ejection, motion, and interaction behavior of multi-channel printing droplets is still required.
(2) Functional ink materials with novel properties have been applied in droplet printing to fabricate specific micro/nano structures [97,98]. However, the generation and motion of droplets during droplet printing occurs under a complex multi-physics field coupling process, easily leading to deformation, fusion, diffusion, breaking-up, etc. Given the need to obtain functional droplets with consistent characteristics, adaptive intelligent control algorithms must be further developed to reduce the attempt cost in terms of both human and material resources.
(3) Composite droplets have exhibited various unexpected performances to promote the application potentials through the interaction effect among different nanomaterials [99,100]. Among them, droplet printing technologies have shown excellent advantages to prepare composite droplets with different liquid channels [101]. However, due to different solution properties like surface tension and conductivity of printing jets in micro/nano scale, the accurate and stable assemble of composite droplets with various dimensions and materials is still a serious challenge requiring concerns.

## Figures and Tables

**Table 1:** Typical methods for droplet printing.

Process Methods	Material	Printing Structures	References
Continuous inkjet printing	Conductive ink	Uniform droplet point	[41]
Continuous inkjet printing	Polyacrylate ink	3D microcircular electrode array	[42]
Continuous inkjet printing	Perovskite materials	High-precision perovskite thin films	[43]
Continuous inkjet printing	Distilled water, n-Octane, n-Tetradecane, and n-Hexadecane	Uniform droplet point	[44]
Thermal inkjet printing	Fibrin	Micron-sized fibrin channels	[45]
Acoustophoretic printing	Newtonian fluids	Microarrays	[46]
Embedded bio-printing	Biological ink	Highly viscoelastic droplets with good circularity	[47]
Piezoelectric inkjet printing	PEDOT:PSS/DMSO/water)	Accurate ink drop point	[48]
Piezoelectric inkjet printing	Conductive ink	Stable droplet array	[49]
Piezoelectric inkjet printing	Conductive ink	Uniform droplet point	[50]
Electromagnetic inkjet printing	Yttria-stabilized zirconia	Electrolyte layers	[51]
Electrohydrodynamic printing	PEDOT:PSS	Stable large-area droplets	[52]
Electrohydrodynamic printing	Conductive ink	High-resolution uniform droplets	[42]
Electrohydrodynamic printing	Ag, CdSe/ZnS	Nanogrids and nanowalls of quantum dots and their composite materials	[53]
Electrohydrodynamic inkjet printing	Conductive ink	Uniform droplet point	[54]
